# SARS-CoV-2 Titers in Wastewater Are Higher than Expected from Clinically Confirmed Cases

**DOI:** 10.1128/mSystems.00614-20

**Published:** 2020-07-21

**Authors:** Fuqing Wu, Jianbo Zhang, Amy Xiao, Xiaoqiong Gu, Wei Lin Lee, Federica Armas, Kathryn Kauffman, William Hanage, Mariana Matus, Newsha Ghaeli, Noriko Endo, Claire Duvallet, Mathilde Poyet, Katya Moniz, Alex D. Washburne, Timothy B. Erickson, Peter R. Chai, Janelle Thompson, Eric J. Alm

**Affiliations:** a Center for Microbiome Informatics and Therapeutics, Massachusetts Institute of Technology, Cambridge, Massachusetts, USA; b Singapore-MIT Alliance for Research and Technology, National University of Singapore, Singapore; c University at Buffalo, The State University of New York, Buffalo, New York, USA; d Center for Communicable Disease Dynamics, Department of Epidemiology, Harvard T. H. Chan School of Public Health, Boston, Massachusetts, USA; e Biobot Analytics, Inc., Cambridge, Massachusetts, USA; f Division of Medical Toxicology, Department of Emergency Medicine, Brigham and Women’s Hospital, Harvard Medical School, Boston, Massachusetts, USA; g Harvard Humanitarian Institute, Cambridge, Massachusetts, USA; h The Fenway Institute, Boston, Massachusetts, USA; i The Koch Institute for Integrated Cancer Research, Massachusetts Institute of Technology, Cambridge, Massachusetts, USA; j Singapore Center for Environmental Life Sciences Engineering, Nanyang Technological University, Singapore; k Asian School of the Environment, Nanyang Technological University, Singapore; l Broad Institute of MIT and Harvard, Cambridge, Massachusetts, USA; m Campus for Research Excellence and Technological Enterprise, Singapore; n Department of Biological Engineering, Massachusetts Institute of Technology, Cambridge, Massachusetts, USA; o Department of Civil and Environmental Engineering, Massachusetts Institute of Technology, Cambridge, Massachusetts, USA; p Selva Analytics, LLC, Bozeman, Montana, USA; University of California San Diego

**Keywords:** COVID-19 prevalence, PMMoV, SARS-CoV-2, viral titers, wastewater

## Abstract

Wastewater surveillance represents a complementary approach to clinical surveillance to measure the presence and prevalence of emerging infectious diseases like the novel coronavirus SARS-CoV-2. This innovative data source can improve the precision of epidemiological modeling to understand the penetrance of SARS-CoV-2 in specific vulnerable communities. Here, we tested wastewater collected at a major urban treatment facility in Massachusetts and detected SARS-CoV-2 RNA from the *N* gene at significant titers (57 to 303 copies per ml of sewage) in the period from 18 to 25 March 2020 using RT-qPCR. We validated detection of SARS-CoV-2 by Sanger sequencing the PCR product from the *S* gene. Viral titers observed were significantly higher than expected based on clinically confirmed cases in Massachusetts as of 25 March. Our approach is scalable and may be useful in modeling the SARS-CoV-2 pandemic and future outbreaks.

**IMPORTANCE** Wastewater-based surveillance is a promising approach for proactive outbreak monitoring. SARS-CoV-2 is shed in stool early in the clinical course and infects a large asymptomatic population, making it an ideal target for wastewater-based monitoring. In this study, we develop a laboratory protocol to quantify viral titers in raw sewage via qPCR analysis and validate results with sequencing analysis. Our results suggest that the number of positive cases estimated from wastewater viral titers is orders of magnitude greater than the number of confirmed clinical cases and therefore may significantly impact efforts to understand the case fatality rate and progression of disease. These data may help inform decisions surrounding the advancement or scale-back of social distancing and quarantine efforts based on dynamic wastewater catchment-level estimations of prevalence.

## Abstract (precis)

Wastewater-based surveillance is a promising approach for proactive outbreak monitoring. SARS-CoV-2 is shed in stool early in the clinical course and infects a large asymptomatic population, making it an ideal target for wastewater-based monitoring. In this study, we develop a laboratory protocol to quantify viral titers in raw sewage via qPCR analysis and validate results with sequencing analysis. Our results suggest that the number of positive cases estimated from wastewater viral titers is orders of magnitude greater than the number of confirmed clinical cases and therefore may significantly impact efforts to understand the case fatality rate and progression of disease. These data may help inform decisions surrounding the advancement or scale-back of social distancing and quarantine efforts based on dynamic wastewater catchment-level estimations of prevalence.

## INTRODUCTION

Improved understanding of the presence and prevalence of SARS-CoV-2 at a population level can help government, public health, and hospital officials implement appropriate policies to mitigate the exponential spread of COVID-19 and diminish the future strain on health care facilities. Despite pandemic spread of SARS-CoV-2 worldwide, broad access to testing in the United States (US) has thus far been severely limited. While it is impractical to test every US resident for SARS-CoV-2, the virus has been found in the stool of confirmed COVID-19 patients (1), making it a promising candidate for wastewater-based surveillance (WBS).

WBS can help detect the presence of pathogens across municipalities and estimate population prevalence without invasive individual testing, and inform public health officials of emerging local hot spots, permitting empirical deployment of testing centers and the efficacy of interventions. The closely related virus SARS-CoV-1 was detected in wastewater from Chinese hospitals during the 2002–2003 SARS pandemic (2), and WBS has been used for the early detection and direct mitigation of disease outbreaks in Israel, Egypt, and Sweden (3–7). We have previously used this technique to measure and map the use of pharmaceuticals across residential communities (8). Here, we describe an analytical technique to detect and quantify genetic material from SARS-CoV-2 in wastewater collected at a large treatment facility.

## RESULTS

We first collected two sewage samples on 18 March 2020 at a major urban wastewater treatment facility in Massachusetts, which has two major influent streams (here named “Northern” and “Southern” influents). Samples were transported to our laboratory where we conducted sample inactivation and viral enrichment (with 80-ml samples), nucleic acid extraction, and RT-qPCR. As negative controls, we used two biobanked wastewater samples (collected on January 8 and 11) from the same treatment facility taken before the first US case was documented.

**FIG 1:**
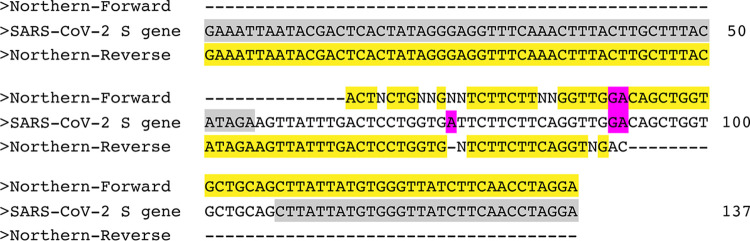
Sanger sequencing results and alignment to SARS-CoV-2 *S* gene. Sequence was aligned to SARS-CoV-2 *S* gene (GenBank MT577641.1) using NCBI BLASTN. Highlighted gray, forward and reverse primers; highlighted yellow, aligned sequence; highlighted purple, mismatched sequence from either primer. The first 24 bases in the COVID-19 *S* gene are the T7 promoter added by PCR with S-F and S-R primers in Table 1. Results for the Southern influent sample were similar. Sequencing chromatograms for forward and reverse primers are provided in Fig. S1B.

Initial testing with PCR and gel electrophoresis using primers specific for the SARS-CoV-2 *S* gene (9) indicated that both samples (from Southern and Northern influents) collected from the treatment facility on 18 March had a positive signal for SARS-CoV-2. We confirmed the signal by identifying a PCR product at 137 bp (see Fig. S1A in the supplemental material). A no-template control for the PCR assays was negative. Sanger sequencing of the PCR products confirmed a 97 to 98% identity match to the SARS-CoV-2 *S* gene (Fig. 1 and Fig. S1B).

10.1128/mSystems.00614-20.1 FIG S1 PCR products with SARS-CoV-2 *S* gene primers and Sanger sequencing results. (A) cDNA was used as the template and run for 42 cycles. Lane 1, DNA ladder (1kb Plus); lane 2, negative control (no template); lane 3, 18 March sample (Southern influent); lane 4, 18 March sample (Northern influent); lane 5, sample on 8 January (combined from Southern and Northern influents); lane 6, sample on 11 January (combined from Southern and Northern influents); lane 7, 50-bp DNA ladder (first three ladders were labeled); lane 8, positive control using cDNA reverse transcribed from synthetic SARS-CoV-2 RNA; lane 9, DNA ladder (1kb Plus). (B) Sanger sequencing results of the PCR product. (Top) Full sequence of the PCR product of *S* gene. Gray nucleotides are the primer sequences. (Middle and bottom) Sequencing chromatograms for forward and reverse primers, respectively. Download FIG S1, EPS file, 2.9 MB. Copyright © 2020 Wu et al. 2020 Wu et al. This content is distributed under the terms of the Creative Commons Attribution 4.0 International license.

We next sought to establish our viral enrichment protocol and quantify viral titer in sewage using RT-qPCR. We used the US CDC primer/probe sets (10) targeting the N1, N2, and N3 loci of the SARS-CoV-2 nucleocapsid gene to amplify cDNA reverse transcribed from viral RNA (Table 1). Standard curves for all three primer sets showed linear behavior over 6 log units (*r*^2^ ranging from 0.981 to 0.998) using DNA standards (SARS-CoV-2 nucleocapsid gene) (Fig. S2). We tested different steps in our viral enrichment process. First, we examined raw (unfiltered) sewage precipitated with polyethylene glycol 8000 (PEG), which recovers both bacterial and viral nucleic acids. Next, we looked at samples taken from 0.2-μm-filtered sewage: we considered both the material collected on the filter and the filtrate. We found the strongest and most consistent results from the PEG-precipitated viral pellet from the 0.2-μm filtrate, which was resuspended in TRIzol for RNA extraction (Table 2).

**TABLE 1:** Primers and probes used in this study^*a*^

Primer	Primer/probe sequence (5′–3′)	Amplicon size (bp)	Target gene	Reference
S-F	GAAATTAATACGACTCACTATAGGGAGGTTTCAAACTTTACTTGCTTTACATAGA	137	Glycoprotein (S)	9
S-R	TCCTAGGTTGAAGATAACCCACATAATAAG			
N1-F	GACCCCAAAATCAGCGAAAT	72	Nucleocapsid (N)	10
N1-R	TCTGGTTACTGCCAGTTGAATCTG			
N1-P	FAM-ACCCCGCATTACGTTTGGTGGACC-BHQ_1			
N2-F	TTACAAACATTGGCCGCAAA	67		
N2-R	GCGCGACATTCCGAAGAA			
N2-P	FAM-ACAATTTGCCCCCAGCGCTTCAG-BHQ_1			
N3-F	GGGAGCCTTGAATACACCAAAA	72		
N3-R	TGTAGCACGATTGCAGCATTG			
N3-P	FAM-AYCACATTGGCACCCGCAATCCTG-BHQ_1			
PMMoV-F	GAGTGGTTTGACCTTAACGTTGA	68	Replication-associated protein	12
PMMoV-R	TTGTCGGTTGCAATGCAAGT			
PMMoV-P	FAM-CCTACCGAAGCAAATG-BHQ_1			

a Shaded primers/probes are used for qPCR. Abbreviations: F, forward; R, reverse; P, probe; FAM, 6-carboxyfluorescein.

**TABLE 2:** Mean *C_T_* values for Southern and Northern samples (18 March 2020) with and without prefiltration (filtrate versus unfiltered) and solids fraction (filter)

Sample	Mean *C_T_* value with primer:		
	N1	N2	N3
Southern-filtrate	33.87	38.39	37.12
Southern-filter	ND^*a*^	ND	ND
Southern-unfiltered	ND	ND	ND
Northern-filtrate	34.91	ND	37.85
Northern-filter	37.86	ND	38.14
Northern-unfiltered	35.04	ND	38.09

a ND, not detected where *C_T_* > 48.

10.1128/mSystems.00614-20.2 FIG S2 Standard curves for N1, N2, and N3 primers. Serial dilutions (10-fold) of a plasmid containing SARS-CoV-2 nucleocapsid gene (*N*) gene were made to generate the standard curves. Value shown is averaged from three replicates. Download FIG S2, TIF file, 5.3 MB. Copyright © 2020 Wu et al. 2020 Wu et al. This content is distributed under the terms of the Creative Commons Attribution 4.0 International license.

These results suggest a simple viral enrichment and RNA extraction protocol is sufficient to achieve viral identification. Importantly, we included pasteurization (90 min at 60°C) as a first step performed before sample containers were opened, increasing the safety of the protocol. Prior work on SARS-CoV-1 indicates that a 30-min heat inactivation at 60°C is sufficient to inactivate the virus by over 6 log units (11). We increased the pasteurization time as an extra precaution for a novel virus and to account for the time required to bring sewage samples to temperature.

**FIG 2:**
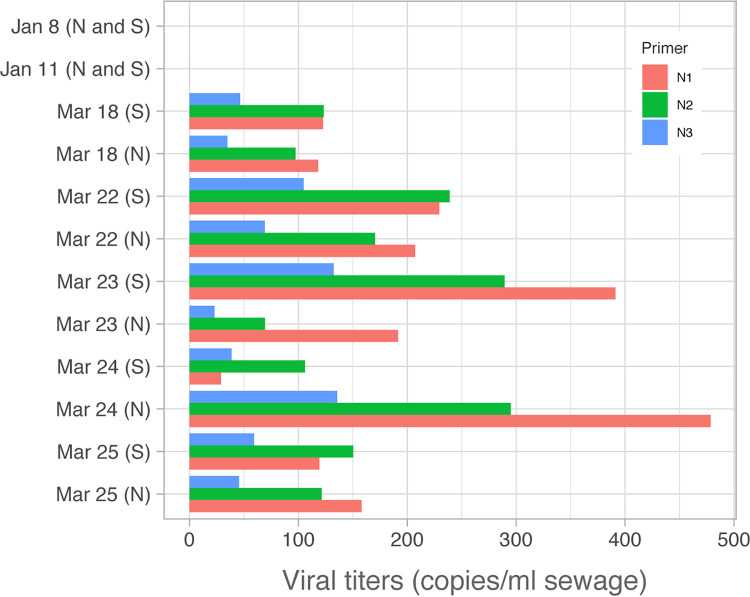
Estimated viral titer per milliliter of sewage. Estimated gene copies in sewage samples for each of the three CDC primer sets are shown for all dates. Northern (N) and Southern (S) influents are shown separately, except for biobanked January samples, which are combined.

After demonstrating the ability of our assay to detect the presence of SARS-CoV-2 in wastewater treatment facility samples, we next quantified the viral titers of SARS-CoV-2 in the wastewater based on the standard curves for each primer set (Fig. S2). Sewage samples from two catchment areas (Southern and Northern) were tested at five sampling dates from 18 to 25 March. All the 10 samples tested were positive for SARS-CoV-2 by RT-qPCR (threshold cycle [*C_T_*] < 40 cycles) (Fig. 2). The mean SARS-CoV-2 titer estimated from all the three primer sets ranges from 57 to 303 copies per ml of sewage. In contrast, both of the biobanked samples collected from before the first known US SARS-CoV-2 case were negative for all three primer sets (Fig. 2).

**FIG 3:**
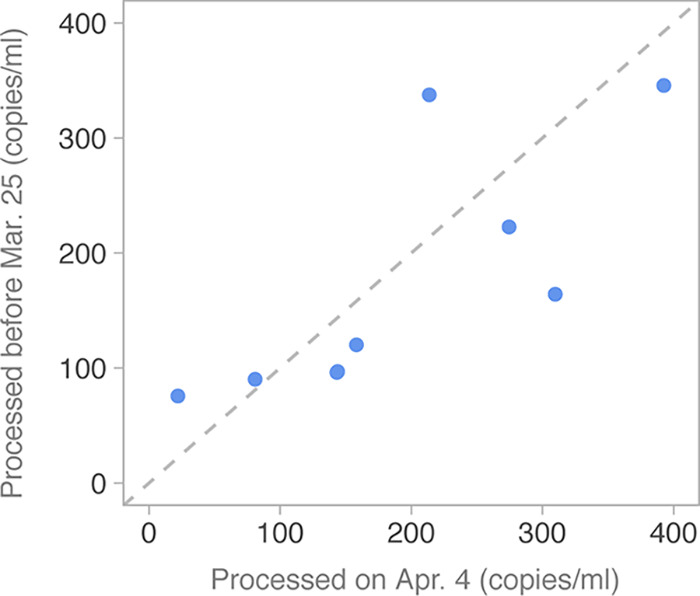
Sample stability over time. Estimated copies per milliliter of sewage estimated from the same samples filtered and processed on different dates. The *y* axis shows samples processed between 20 and 25 March 2020, and these are plotted against samples reprocessed on 4 April 2020 (samples were pasteurized upon receipt and stored at 4°C until processing). The gray dashed line corresponds to *y* = *x*. Each point represents an average over all three primer sets for each sample.

Next, we examined how quickly viral signal degraded during storage at 4°C. All samples were received during 20 to 25 March, processed upon initial receipt, and then stored at 4°C. We reprocessed those samples starting from pasteurized sewage on 4 April. Figure 3 shows that there was variation between sample runs on different dates, but no clear trend toward lower signals at the later time point, suggesting samples can be stored at 4°C for more than a week (9 to 15 days) without significant degradation of viral RNA in pasteurized sewage samples.

Viral titers in sewage samples are determined by the concentrations of fecal materials in the total flow at the wastewater treatment plant. However, the sewage flow rate is not stable and is impacted by many factors including inflows from rainstorms. This factor is prominent especially during this dynamic winter-spring season in Massachusetts. To correct those variations, we use pepper mild mottle virus (PMMoV) as an internal reference for quantification of SARS-CoV-2. Previous studies have shown that PMMoV is the most abundant RNA virus in human feces and it is shed in large quantities in wastewater across the United States (12–16). PMMoV is remarkably stable in the wastewater, and its concentrations showed little seasonal variation (15, 16). Furthermore, like SARS-CoV-2, PMMoV is also a positive-sense single-stranded RNA virus, making it suitable as an internal reference to help control for sample-to-sample variability in wastewater dilution or processing. These properties allow us to calibrate the SARS-CoV-2 titers across the samples.

**FIG 4:**
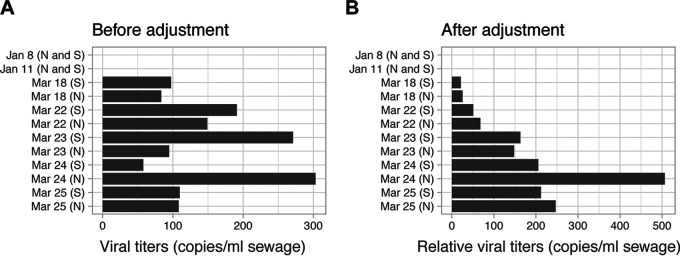
PMMoV normalization for sewage samples collected from 18 March to 25 March. (A) Original SARS-CoV-2 titers averaged from N1, N2, and N3 primers in the sewage sample. (B) Relative SARS-CoV-2 titers against PMMoV concentration in each of the samples. Data shown are averaged from N1, N2, and N3 primers. Influent samples from Northern (N) and Southern (S) influents are shown separately for each date.

The qPCR results showed that PMMoV levels varied across the 12 samples, with a standard deviation of 1.34 *C_T_* units. We normalized our data in Fig. 2 based on the PMMoV concentration in each sample and its deviation from the median value. Figure 4 shows that the data after normalization are much less noisy and match the upward trend of clinical COVID-19 cases reported during 18 to 25 March (17). Thus, these data suggest that PMMoV may be useful as an internal reference to reduce sample variance.

These data suggest an order of magnitude estimate of approximately 100 viral genome copies per ml of sewage where titers in wastewater varied from 57 to 303 copies/ml, and from 21 to 506 copies/ml after PMMoV normalization. From these findings and analyses, we can draw the following deductions concerning disease prevalence. First, we note that any rigorous conclusions depend on a number of factors that are unknown, such as the timeline and load of fecal shedding, loss of viral particles in sewage lines, and the exact loss of viral RNA during experimental procedures, and thus, additional experiments will be required to calibrate these numbers. Nonetheless, we can estimate an abundance based on the lowest observed values across these samples of ∼10 copies/ml. If we assume typical stool sizes of 200 g, diluted into an average daily flow volume of 1.36 × 10^9^ liter, and a population of 2.3 × 10^6^ individuals each producing one stool per day, and we further assume that there is no loss of viral RNA in sewer lines and that excreted viruses are fully suspended in sewage, then we expect the viral titer in feces to be about 3,000 times higher than that in sampled raw sewage, or about 30,000 particles per ml.

Estimates of viral load in stool from positive patients are still a matter of uncertainty, where median levels of 12,882 copies/g (18) reflect a range from nondetection of SARS-CoV-2 RNA in some stool samples and levels as high as 600,000 to 30,000,000 viral genomes per ml or g of fecal material in other samples (18, 19). Estimating an average of 600,000 viral genomes per ml in stool (19) would suggest that roughly 5% of all fecal samples in the treatment facility catchment were positive for SARS-CoV-2 in the 18 to 25 March period, a number much higher than the 0.026% confirmed for the state of Massachusetts (a similar prevalence is obtained using individual counties represented by the wastewater treatment facility’s catchment or statewide estimates) on 25 March (17). Similarly, taking the extreme of stool concentration of 30 million copies/ml (18) suggests that we would have a prevalence of 0.1%, closer to, but still higher than, the number of confirmed clinical cases. Additional data on viral shedding in stool over the clinical course of the disease in patients with documented SARS-CoV-2 may be required to better interpret these findings.

## DISCUSSION

Here, we established and verified a protocol to detect and quantify SARS-CoV-2 titers in wastewater samples. Starting with pasteurization, raw sewage samples were first filtered and precipitated with PEG-NaCl for viral enrichment, followed by RNA extraction, reverse transcription, and real-time PCR with SARS-CoV-2-specific primers. With this method, we detected SARS-CoV-2 in all of the 10 samples from 18 to 25 March in the influents of a wastewater treatment plant in Massachusetts. This simple protocol does not rely on expensive chemicals or materials and thus could be widely employed for viral detection in wastewater samples. Furthermore, we avoided the competitive usage of commercial kits like viral RNA extraction kits and one-step RT-PCR enzyme master mix kits that were recommended by the CDC protocols widely used in clinical testing (10).

Viral titers were quantified based on standard curves for the three primer sets (see Fig. S2 in the supplemental material). However, a complete quantification of viral titers needs further investigation of recovery rates for each step including the pasteurization, filtering, viral precipitation, RNA extraction, and reverse transcription. Recently, Ahmed et al. used the beta-coronavirus murine hepatitis virus (MHV) as a closely related surrogate for SARS-CoV-2 to quantify the recovery rates from wastewater; using various methods including PEG precipitation, they found that MHV recoveries ranged from 26.7 to 65.7% (20). To adjust the variations from the initial sample concentrations and RNA losses during these experimental procedures, we used PMMoV, the most abundant RNA virus in human stool (13, 16), as a reference. Our results showed that the PMMoV concentrations varied across the 12 samples with a standard deviation of less than 1.34 units, which also fall in the variation range of PMMoV concentrations in different seasons in a previous study (15). However, studies with more wastewater samples over a longer period are needed to understand whether the PMMoV loading is stable over time for the catchment population.

This discrepancy between confirmed cases and observed viral titers could arise from a number of factors. First, our estimates are based on numbers and assumptions that are currently subject to significant uncertainty, as discussed above, and therefore could be inaccurate. However, we note that our calculations are conservative because we (i) apply the lower limit of our observed viral titers in wastewater; (ii) assume no loss in viral titer due to degradation, sample processing, or RNA extraction; and (iii) consider high viral loads per infected stool, thus requiring fewer positive stools to generate the observed wastewater titers. The estimates of viral titer per positive stool may be too low if a small number of individuals shed very high levels of virus. While this can be determined, it will require more extensive testing of individual stool specimens from patients with documented COVID-19 disease in order to arrive at an accurate estimate. Given the importance of assessing the fraction of SARS-CoV-2 infections that present with symptoms, we note that our results are consistent with the idea that a significant fraction of cases are not detected with current testing algorithms, and that this fraction may include a large number of asymptomatic individuals (21, 22). Limitations of testing capacity and stringent criteria for testing would lead to an underestimate of clinically confirmed cases, whereas wastewater-based surveillance provides an anonymous, unbiased sample of the entire infected population, potentially explaining the magnitude of the difference we observe.

To discriminate between these possibilities, additional catchments of different sizes should be tested. By integrating data on the presence or absence of viral particles across catchments of varying size within the same geographic region, it may be possible to estimate disease prevalence independent from knowing the average viral titer in infected stool. For example, if disease prevalence is 1 in 10,000, then approximately 50% of catchment areas representing ∼7,000 individuals would be positive (although cases may cluster within households, so additional experiments or modeling would be needed to derive precise numbers). These experiments require sampling upstream areas in the sewage system, using specialized equipment to strategically capture time-integrated samples, and are under way. Once these experiments are completed in a small number of regions, they could be used to estimate disease prevalence in other regions using wastewater treatment facility data alone.

### Conclusions.

These data demonstrate the feasibility of measuring SARS-CoV-2 in wastewater using a method that does not require materials that are in high demand for individual clinical testing. The implications of this research are that wastewater-based surveillance can be leveraged to detect population-level prevalence of SARS-CoV-2 in cities and municipalities across the world. In a setting where in-person testing may not be available or may be overly cost-prohibitive, longitudinal analysis of wastewater can provide population-level estimates of the burden of SARS-CoV-2.

These data may help inform decisions surrounding the advancement or scale-back of social distancing and quarantine efforts based on wastewater catchment-level estimations of prevalence. Additionally, wastewater collection at the municipal or community level may allow for more granular detection of SARS-CoV-2 in cities with lower COVID-19 disease burden, thereby functioning as an early warning system to help preemptively enact public health measures prior to the widespread onset of disease.

## MATERIALS AND METHODS

### Sewage samples and pasteurization.

Twenty-four-hour composite samples (1 liter) of raw sewage from 18 to 25 March were taken from a major urban wastewater treatment facility in Massachusetts. Those samples are from two catchment areas (Southern and Northern influents) except biobanked samples on 8 and 11 January, which were a mixture of Southern and Northern influents. In total, we collected 12 wastewater samples including 2 samples in January (8 and 11 January) and 10 samples from 18 to 25 March. Upon initial receipt, samples were placed in the biosafety cabinet with UV for 20 min and then pasteurized in a 60°C water bath for 90 min to inactivate the virus. Previous studies showed that pasteurization could effectively inactivate the virus without compromising sample quality (23, 24). Pasteurized samples were then used for viral precipitation, and the remaining samples were stored at 4°C.

### Prefiltration evaluation.

We tested the SARS-CoV-2 signals in the unfiltered samples, filtrate collected by filtration through a 0.22-μm polyether sulfone membrane (catalog no. SCGP00525; Millipore Sigma), and solid materials on the filter, respectively. Unfiltered sewage samples and filtrate were then precipitated with polyethylene glycol 8000 and NaCl and processed as described below. Solid materials on the filter were directly resuspended with 1.5 ml TRIzol and lysed for 10 min at room temperature. Undissolved materials were removed by centrifugation before the RNA extraction steps. The strongest and most consistent SARS-CoV-2 signal was detected from the PEG-precipitated filtrate fraction of the Northern and Southern samples, which was higher than the signal from the unfiltered samples or the solid fraction. No viral signal was detected in the solid fraction on the filter for the Southern sample, whereas only ∼10% of SARS-CoV-2 signal for N1 primer was found on the solid materials on the filter for the Northern sample (Table 2). Thus, we concluded that there was little viral RNA on the filters and used the filtrate for detection.

### Viral precipitation.

Pasteurized sample was first filtered through a 0.2-μm membrane (catalog no. SCGP00525; Millipore Sigma) to remove bacterial cells and debris. For the initial test, we used 80 ml filtrate of two samples on 18 March (Southern and Northern) for the viral precipitation with polyethylene glycol 8000 (8% [wt/vol]; Millipore Sigma) and NaCl (0.3 M; Millipore Sigma). Samples were shaken at room temperature for about 15 min (until the chemicals were fully dissolved) and then centrifuged at 12,000 × *g* for 2 h or until a pellet was visible. The viral pellet was then resuspended in 1.5 ml TRIzol reagent (ThermoFisher) for RNA extraction. After validation of this method (Fig. 1 and Table 2), we used 40 ml filtrate for the viral precipitation and processed all the 12 samples in the same way.

### RNA extraction and RT-qPCR.

RNA was extracted from the filtered sewage samples using the TRIzol-chloroform approach. Briefly, samples resuspended in the TRIzol reagent were thoroughly mixed with 300 μl chloroform for 1 min and incubated at room temperature for 5 min before centrifugation (16,000 × *g*, 15 min, 4°C). Six hundred microliters of aqueous phase was transferred to a new 1.5-ml tube and thoroughly mixed with an equal volume of isopropanol. After centrifugation at 16,000 × *g* for 10 min, the supernatant was discarded and the pellet was washed twice with 75% ethanol. Thirty microliters of diethyl pyrocarbonate (DEPC) water was used to recover the RNA.

cDNA was synthesized by the reverse transcription with random hexamers (IDT) based on manufacturer’s protocol (NEB catalog no. M0368). PCR includes TaqMan Fast Advanced master mix (4444557; ThermoFisher Scientific); CDC N1, N2, and N3 primer-probes (IDT); and cDNA as a template. The qPCR was carried out for 48 cycles on a Bio-Rad CFX96 real-time PCR detection system (Bio-Rad) based on the following program: polymerase activation (95°C for 2 min) and PCR (48 cycles, denaturation at 95°C for 1 s, and annealing/extension at 55°C for 30 s). Three replicates were performed for each primer, mean values were reported, and the coefficient of variation between replicates was <8%. The positive control for qPCR and construction of the standard curve for N1, N2, and N3 assays was a plasmid (initial concentration: 2 × 10^5^ copies/μl; IDT catalog no. 10006625) containing the complete nucleocapsid gene from SARS-CoV-2.

Output from the qPCR assay for the pepper mild mottle virus (PMMoV) (primers shown in Table 1) was used to calibrate the SARS-CoV-2 titers. To adjust the SARS-CoV-2 viral titers for each sample, we first calculated the deviation of its PMMoV copies from the median of PMMoV copies in all samples, i.e., deviation factor = 10̂[*k* × (sample *C_T_* − median *C_T_*)], where *k* is the slope of the standard curve and equals −0.2991 (amplification efficiency is 99.11% for the PMMoV primer set) based on our test. We then divided the SARS-CoV-2 viral titers by this deviation. We noted that viral titers for 24 March samples (Southern and Northern) increased by 1.6- to 3.5-fold after PMMoV adjustment (Fig. 3). This is because the PMMoV concentrations for the two samples were lower than the median of PMMoV copies in the 12 sewage samples, suggesting that the two samples were more dilute than the other samples. It is interesting that there was significant precipitation in the Boston area during 23 to 24 March (https://www.timeanddate.com/weather/). The inflows from snow and heavy rainfall might explain the diluted wastewater samples on 24 March.

### PCR, gel electrophoresis, and Sanger sequencing.

To test SARS-CoV-2 identity in the wastewater samples and avoid potential contamination from *N* gene positive control (IDT catalog no. 10006625), we amplified an *S* gene fragment using primers shown in Table 1. PCR was performed with KAPA Hi-Fi Hot Start ready mix 2× (Fisher Scientific; KK2602), and cDNA was used as the template with the following program: 95°C for 5 min and 95°C for 30 s, 55°C for 30 s, and 72°C for 20 s for 42 cycles. PCR products were run on a 2% precast agarose E-gel (ThermoFisher; G402002). The positive control was cDNA reversed transcribed from synthetic SARS-CoV-2 RNA (Twist catalog no. 102024). PCR products were sent to Genewiz (Cambridge, MA) for purification and Sanger sequencing with forward and reverse primers (Table 1), and low-quality sequence adjacent to sequencing primers and terminal N’s were removed. Sequencing results were subjected to BLAST search with NCBI BLASTN (https://blast.ncbi.nlm.nih.gov).
