# Supplementary figures and images for: SARS-CoV-2 Titers in Wastewater Are Higher than Expected from Clinically Confirmed Cases
Source: mSystems. 2020 Jul 21;5(4):e00614-20. doi: 10.1128/mSystems.00614-20 (PMC7566278; doi:10.1128/mSystems.00614-20)

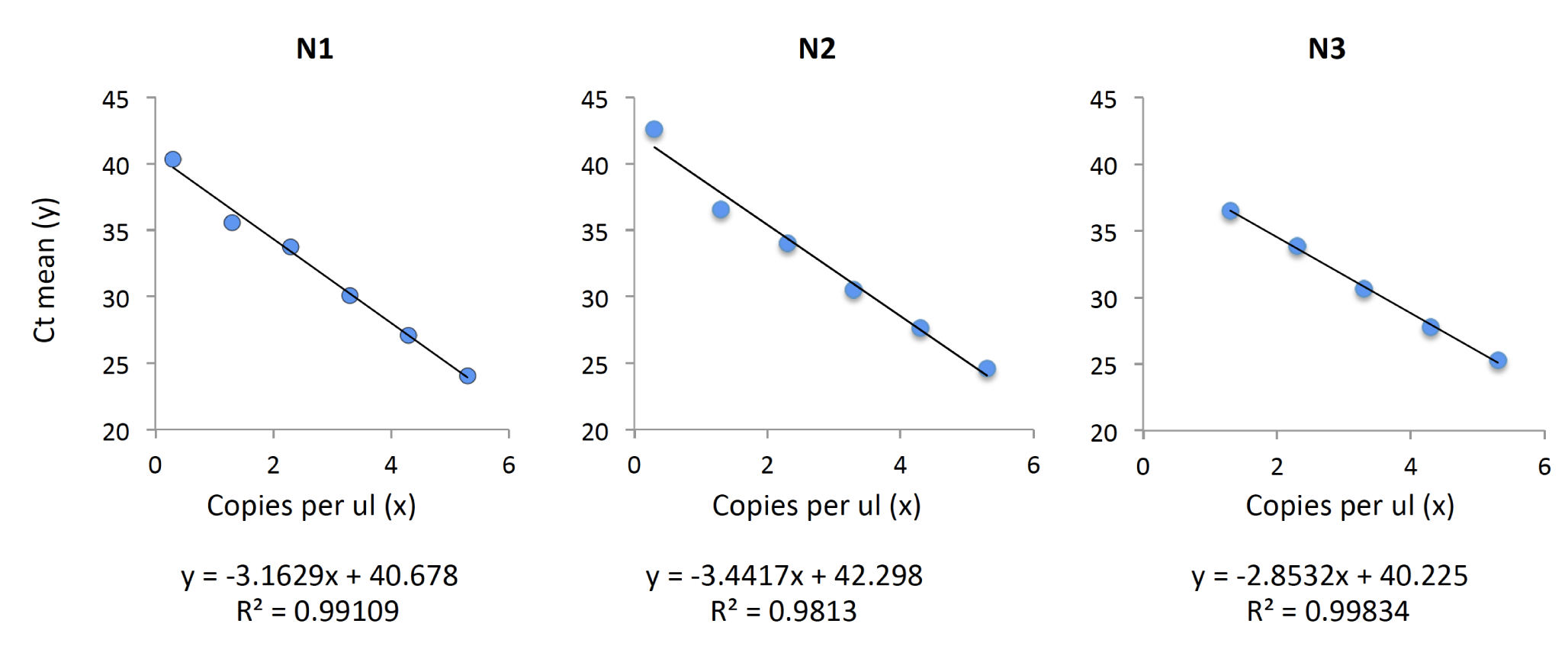

Supplement: FIG S2 [file mSystems.00614-20-sf002.tif]
